# A large *Cryptosporidium parvum* outbreak associated with a lamb-feeding event at a commercial farm in South Wales, March–April 2024: a retrospective cohort study

**DOI:** 10.1017/S0950268825100198

**Published:** 2025-07-14

**Authors:** Gethin Jones, Joshua Matizanadzo, Andrew Nelson, Rachel M. Chalmers, Daniel Rhys Thomas, Stuart Williams, Maria Pinch, Alison Sykes, Rhianwen Stiff, Chris Williams

**Affiliations:** 1 UK Field Epidemiology Training Programme, UK Health Security Agency, London, UK; 2 Communicable Disease Surveillance Centre, Public Health Wales, Cardiff, UK; 3 Cryptosporidium Reference Unit, Public Health Wales, Swansea, UK; 4 Food Safety, Health and Safety and Communicable Disease Control Team, Caerphilly County Borough Council, Caerphilly, UK; 5 Health Protection Team, Public Health Wales, Cardiff, UK

**Keywords:** Cryptosporidium, cryptosporidiosis, lamb-feeding, farm, cohort study, outbreak

## Abstract

*Cryptosporidium parvum* is a well-established cause of gastrointestinal illness in both humans and animals and often causes outbreaks at animal contact events, despite the availability of a code of practice that provides guidance on the safe management of these events. We describe a large *C. parvum* outbreak following a lamb-feeding event at a commercial farm in Wales in 2024, alongside findings from a cohort study to identify high-risk exposures. Sixty-seven cases were identified, 57 were laboratory-confirmed *C. parvum*, with similar genotypes. Environmental investigations found a lack of adherence to established guidance. The cohort study identified 168 individuals with cryptosporidiosis-like illness from 540 exposure questionnaires (distributed via email to 790 lead bookers). Cases were more likely to have had closer contact with lambs (odds ratio (OR) kissed lambs = 2.4, 95% confidence interval (95% CI): 1.2–4.8). A multivariable analysis found cases were more likely to be under 10 years (adjusted OR (aOR) = 4.5, 95% CI: 2.0–10.0) and have had visible faeces on their person (aOR = 3.6, 95% CI: 2.1–6.2). We provide evidence that close contact at lamb-feeding events presents an increased likelihood of illness, suggesting that farms should limit animal contact at these events and that revisions to established codes of practice may be necessary. Enhancing risk awareness among farmers and visitors is needed, particularly regarding children.

## Background

*Cryptosporidium* is a highly infectious protozoan parasite transmitted by ingestion of infectious oocysts via the faecal–oral route. Cryptosporidiosis is a well-established cause of gastrointestinal illness in humans and livestock, although there is evidence that it is both underdiagnosed and underreported [1]. Symptoms typically include watery diarrhoea, abdominal pain, vomiting, loss of appetite, and a mild fever [2]. Cryptosporidiosis is usually self-limiting, but in some groups, particularly young children and the immunosuppressed, illness can be severe with long-term health and societal impacts [3–5]. Even in non-immunocompromised people, symptoms can persist beyond the acute phase of the illness [6]. The two predominant species in human cryptosporidiosis are *Cryptosporidium hominis*, which is predominantly anthroponotic, and *C. parvum*, which is zoonotic; both of which display seasonal trends [1]. In the United Kingdom, *C. hominis* case numbers and outbreaks peak in autumn, and *C. parvum* peaks during spring [7, 8]. Reporting of both species declined during the coronavirus disease 2019 pandemic period but has since rebounded, with *C. parvum* recovering more rapidly than *C. hominis* [9].

*C. parvum* is found in young livestock, including lambs, and is often associated with human outbreaks in farm settings [10–12], particularly those offering lamb-contact events [13]. One recent analysis found that, between 2009 and 2017, almost half (42%) of human cryptosporidiosis outbreaks in England and Wales were associated with animal contact [8]. Given the rising popularity of commercial farms offering public access to livestock in the United Kingdom, especially during lambing or through the increasing popularity of schemes such as ‘Open Farm Sunday’ [14], and the emergence of social media and online booking to promote these events, there is an increased risk of exposure to *C. parvum* [12].

A code of practice [15] was published in 2021 by the industry in consultation with the Health and Safety Executive to provide guidance and control measures covering how to safely operate and manage animal-contact events at UK farms offering public access. This guidance highlights the need to carry out site-specific risk assessments, quarantine sick animals, the importance of appropriate staff supervision, and ensuring handwashing facilities are clean, adequate, accessible, and their use encouraged, alongside ensuring animal contact areas remain free from faeces, and that eating areas are segregated from any areas designated for animal contact.

In March 2024, several cases of laboratory-confirmed *Cryptosporidium* spp. were reported to Public Health Wales (PHW) in people with links to a commercial farm (Farm X) offering a variety of public events, including a lamb-feeding experience. An Incident Management Team (IMT) was convened on 2 April to investigate the outbreak and inform public health action. Environmental, epidemiological, and microbiological investigations were instigated, with the primary hypothesis that illness was associated with the lamb-feeding experience offered at the farm. A retrospective cohort study was also undertaken to investigate factors associated with illness at the farm and to provide recommendations for the prevention and management of future outbreaks.

Farm X voluntarily closed the barn and lamb-feeding experience to the public following the report of the first cases on 26 March 2024, and the owner requested a veterinary visit to assess the lambs.

## Methods

### Outbreak investigation

Following the declaration of the outbreak on 5 April 2024, case definitions were agreed (Table 1) and case finding continued through routine surveillance of laboratory-diagnosed *Cryptosporidium* cases and reports of cases and/or diarrhoea to local health protection teams.

**Table 1.** Case definitions used for the original outbreak investigation and subsequent cohort study

Outbreak investigation	
Confirmed case	Any person who:
	Visited Farm X since 1 February 2024
	AND
	Developed gastrointestinal symptoms^a^ within 3–14 days of attendance
	AND
	Has a local laboratory confirmation of *Cryptosporidium*
Probable case	Any person who:
	Visited Farm X since 1 February 2024
	AND
	Developed gastrointestinal symptoms^a^ within 3–14 days of attendance
Confirmed secondary case	Any person who: Was a close contact (household or similar) of someone who visited Farm X since 1 February 2024 AND Developed gastrointestinal symptoms^a^ within 3–14 days of contact AND Has a local laboratory confirmation of *Cryptosporidium*
Probable secondary case	Any person who: Was a close contact (household or similar) of someone who visited Farm X since 1 February 2024 AND Developed gastrointestinal symptoms^a^ within 3–14 days of contact

Cohort study	
Probable	Any person who:
	Attended Farm X between 1 March 2024 and 26 March 2024
	AND
	Developed gastrointestinal symptoms^a^ within 3–14 days of attendance
Non-case	Any person who:
	Attended Farm X between 1 March 2024 and 26 March 2024
	AND
	Did not have gastrointestinal symptoms^a^

a Gastrointestinal symptoms include diarrhoea, vomiting, nausea, abdominal pain, and fever.

### Microbiology

Cases were confirmed in local diagnostic microbiology laboratories by polymerase chain reaction (PCR) or enzyme immunoassay, with positive stool samples sent to the Cryptosporidium Reference Unit (CRU) in Swansea for species identification by real-time PCR and subtyping by gp60 sequencing and multi-locus tandem repeat variable analysis (MLVA) [16–18].

Faecal samples were taken from lambs from different pens in the barn for diagnostic purposes by the private veterinary surgeon, most of which tested positive for *Cryptosporidium* spp. by immunochromatographic lateral flow test and modified Ziehl–Neelsen stained microscopy. One of these positive samples was available and sent for *Cryptosporidium* species identification and subtyping at the CRU with support from the Animal and Plant Health Agency (APHA). Samples were not obtained from the goats or calves in the barn.

### Environmental investigations

Local Environmental Health Officers (EHOs) led environmental investigations during the outbreak and visited the farm on three separate occasions between 28 March and 24 April to review infection prevention and control measures in place on the site, alongside discussions of those events open to the public. An additional site visit by EHOs and PHW took place on 22 April to review the site and gain further contextual information about the site and event.

### Cohort study

We undertook a cohort study to clarify which exposures lead to illness in farm visitors. The study population was defined as any individual who attended Farm X between 1 March 2024 and 26 March 2024. Study case definitions are provided in Table 1.

We used a list of individuals received from the farm containing the contact information of those who had booked to attend the lamb-feeding experience to recruit for the study. All individuals in the list were sent a link to an online questionnaire (Supplementary Material 1) by email. As these bookings may have been for more than one individual, we asked that the questionnaire be forwarded to other adults in their party, with parents/guardians of children asked to submit responses on their child’s behalf.

The questionnaire included questions on demographics, symptoms, date of onset, healthcare seeking behaviour, and farm exposures (including risk and protective behaviours, such as level of animal contact, whether they ate/drank on site, and hand hygiene). Individuals were excluded from the study if they stated they did not attend the farm, had a symptom onset of fewer than 3 days (i.e., possible background cases) or more than 14 days following attendance (i.e., possible secondary cases), or if respondents disengaged with the survey having only completed basic demographic information.

In order to identify on-farm risks for infection, we carried out univariable analysis to calculate odds ratios (ORs) and 95% confidence intervals (95% CIs) comparing illness outcome against various exposures. Stratified analysis was undertaken to examine potential confounding and effect modification. As we could not be assured that all visitors to the farm were contacted and received a questionnaire, we used ORs instead of risk ratios as the measure of association.

A multivariable logistic regression model was constructed using forward stepwise inclusion of variables (*p*-value < 0.2), added in order of significance, and considering goodness of fit (Akaike information criterion) to obtain adjusted ORs (aORs) and 95% CIs. Variables with co-linearity and those that provided little improvement in the Akaike information criterion were dropped from the final model, along with those variables with a high proportion of missing values.

An additional dose–response analysis was also conducted to examine outcomes among those exposed to varying levels of lamb contact during the event. The level of contact was grouped into four categories, from lowest to highest, as follows: (1) fed lamb without touching or petting without holding; (2) held lamb on lap; (3) cuddled lamb; and (4) kissed/nuzzled face with lamb, with the lowest level of contact used as the baseline for comparison.

Data processing and analysis were performed in R (version 4.1.3) [19]. Stratified analysis was performed in STATA SE (version 14.2) [20].

## Results

### Description of the outbreak

**Figure 1.**
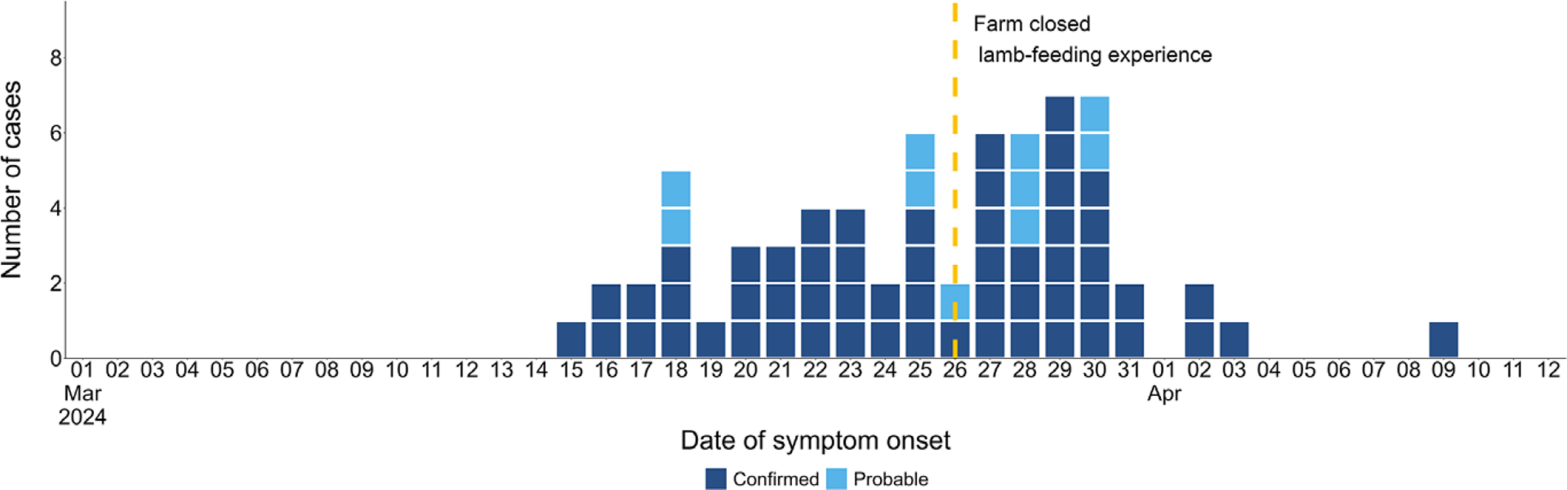
Number of notified cases identified by the Incident Management Team by date of symptom onset and case definition. Also showing the date Farm X closed the lamb-feeding experience.

The outbreak investigation identified 67 cases (57 confirmed and 10 probable) residing in 9 local authorities in South Wales (Figure 1). Four of the 67 cases (2 confirmed and 2 probable) were considered secondary cases. The age range for cases was 1–62 years, with a high proportion of children aged 0–9 years (33%) and adults aged 30–39 years (30%); the majority of cases were female (69%). Diarrhoea was the most commonly reported symptom (90%), followed by abdominal pain (76%) and vomiting (69%). Of the cases identified during the initial outbreak investigation, 18 (27%) attended the hospital for their symptoms; of these, 10 (15% of all cases) were admitted to the hospital overnight with an average stay of 2.2 nights. The median age of those admitted to the hospital was 25 years (range 1–36).

The outbreak was declared over on 26 April 2024

### Microbiology

Of the 57 outbreak case specimens referred to the CRU, 56 were identified as *C. parvum.* Two closely linked MLVA profiles (4-13-6-7-27-21-16 and 4-13-6-7-27-21-15) and one mixed profile (4-13-6-7-27-21-15/16) were found in 26, 6, and 21 case specimens, respectively. Four specimens were reported with incomplete MLVA profiles.

A single gp60 subtype, IIaA15G2R1, was identified in the initial 23 specimens tested. Further specimens were not tested by sequencing the *gp60* gene, as the MLVA profiles were consistent.

A faecal sample retrieved from one of the lambs used for the event was found to be *C. parvum*, with a mixed MLVA profile (4-13-6-7-27-21-15/16) and gp60 subtype IIaA15G2R1, providing a match to those reported among human cases.

### Environmental investigations

Discussions with the farm owner revealed that ~50 lambs were included in the lamb-feeding experience. Half of these were imported from two local farms for the event: five from Farm Y on 23 March and 20 from Farm Z on 24 March. The remainder were from the farm’s own stock. The lamb-feeding event was promoted on the farm’s Facebook page to boost attendance. This featured photographs of the lambs and visitors feeding them, with the aim of attracting families. Tickets could be booked online and were sold on a group basis, meaning a booking could include a single person or multiple people. Each booking was allocated a bottle to feed the lambs. The farm owner estimated that each bottle would be used by approximately two to three people. Lambs were housed in a barn near the farm entrance and, on arrival, visitors would be brought into the barn, and each booking group was handed a lamb and a bottle to feed them. The barn housing the lambs also housed a smaller number of goats and calves, which remained penned for the event but could be petted and hand fed (using dry pellets) by visitors.

Site inspections of the farm revealed handwashing facilities to be rudimentary, with one sink available at the entrance/exit of the barn, which was supplied by cold water only. Picnic tables were set up in the barn for attendees, with a small number of signs highlighting the importance of handwashing visible. Faeces were present on the barn floor despite being recently cleaned, suggesting this was also likely during the event. The farmer reported to EHOs inspecting the premises that visitors often held the lambs for prolonged periods, during which the lambs often urinated or defecated on visitors.

Case interviews indicated that the paper towels and soap provided had often run out, and that some lambs seemed unwell. Social media photos of the event indicated a close level of contact with lambs, as well as visitors eating and drinking at the picnic tables in the barn.

### Cohort study

Four hundred twenty-one of the 790 lead bookers contacted responded to the survey (53.3%). A further 472 individuals were recruited from the booking groups, which in total generated 893 individual questionnaire responses after de-duplication (61 removed), with an average of 2.1 responses per booking. In total, 82 individuals were excluded due to not attending the farm during the period of interest, 205 were excluded for non-completion of key data fields (such as farm exposures and symptom information), and a further 66 were excluded as they did not meet the case definition in terms of symptom onset (3–14 days post exposure), leaving a study population of 540 for the final analysis.

**Figure 2.**
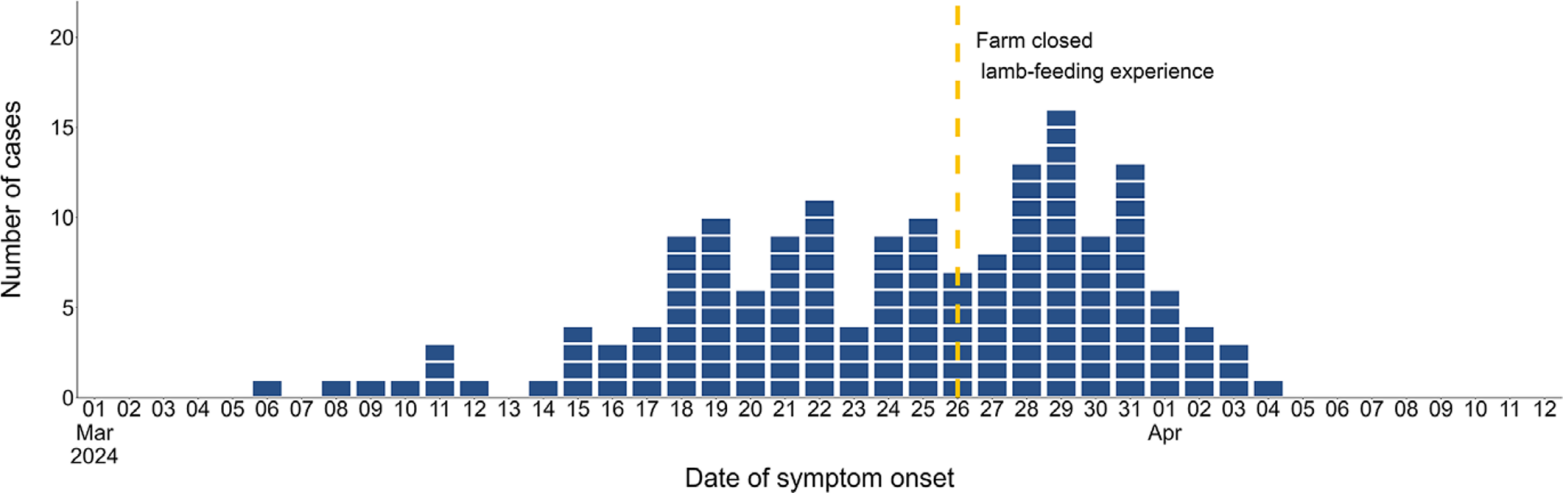
Number of cases included in the cohort study by date of symptom onset. Also showing the date Farm X closed the lamb-feeding experience.

**Figure 3.**
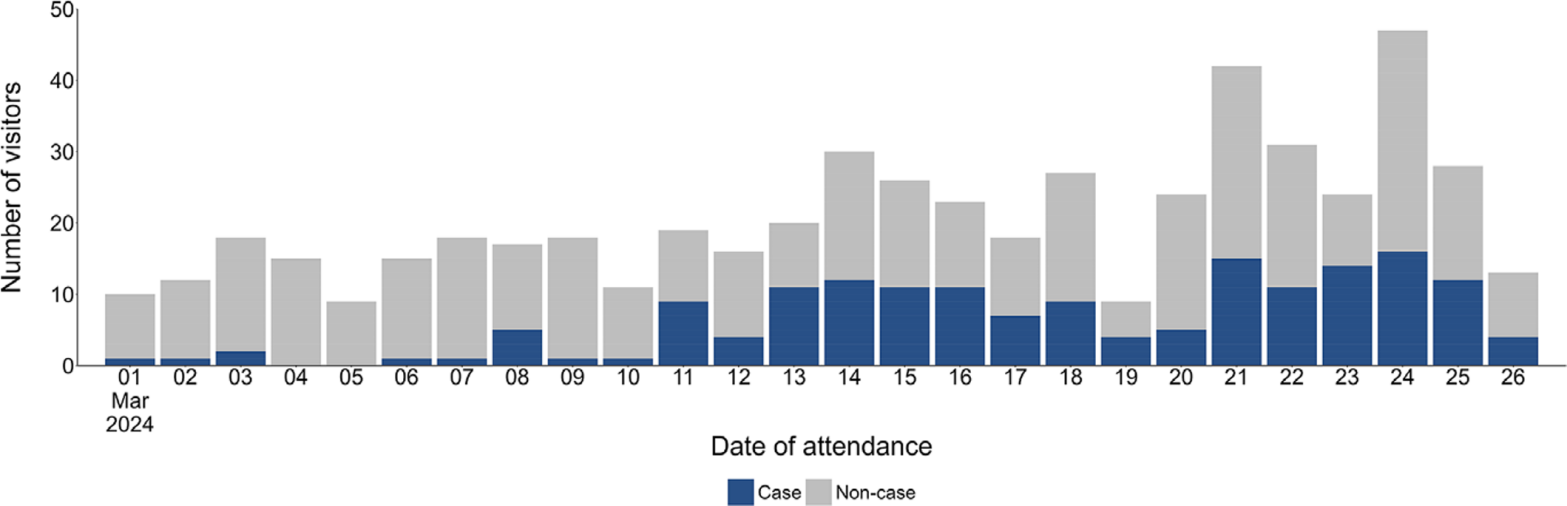
Number of visitors to Farm X by date of attendance and case status.

Of the 540 people recruited into the cohort study, 168 met the case definition with symptom onset dates ranging between 6 March and 4 April 2024 (Figure 2). Thirty-three cases were known as part of the initial outbreak investigation (31 confirmed). The median incubation was 6 days, and the median symptom duration was 8.5 days (range 0–51). For those still experiencing symptoms at the time of completing the survey, the survey completion date was taken as a proxy to indicate minimum symptom duration. The number of people reporting symptoms following attendance at the farm (cases) increased from the 11 March, with the highest proportion of cases attending on the 24 March 2024 (Figure 3).

The majority of the 168 cases identified in the cohort were female (79.2%), and ages ranged between 1 and 80 years (Table 2). A higher proportion of cases were aged under 10 years (15.6%) compared to non-cases (7.3%). Diarrhoea (93.5%), watery diarrhoea (86.9%), and abdominal pain (69.6%) were the most common symptoms reported, all of which are consistent with cryptosporidiosis. In terms of severity, 63 (37.5%) cases reported seeking healthcare for their symptoms, including 18 (10.7%) who presented to the hospital. Seven cases were admitted overnight with an average stay of 2.4 nights. The median age of those admitted was 19 years (range 1–36), slightly younger than that described in the outbreak investigation.

**Table 2.** Characteristics of the cohort study population

	Case		Non-case	
	n	%	n	%
	168	31%	372	69%
Age group (years)				
0–9	26	15.6%	27	7.3%
10–19	12	7.2%	20	5.4%
20–29	23	13.8%	45	12.2%
30–39	56	33.5%	139	37.7%
40+	28	16.8%	113	30.6%
Unknown	22	13.2%	25	6.8%
Sex				
Male	32	19.0%	72	19.4%
Female	133	79.2%	291	78.2%
Unknown	3	1.8%	9	2.4%
Symptoms				
Diarrhoea	157	93.5%		
Watery diarrhoea	146	86.9%		
Abdominal pain	117	69.6%		
Loss of appetite	99	58.9%		
Nausea	98	58.3%		
Vomiting	80	47.6%		
Fever	53	31.5%		
Sought healthcare				
Yes	63	37.5%		
Healthcare seeking behaviour (not exclusive)				
General Practice (GP)	44	26.2%		
NHS Direct	20	11.9%		
Accident and Emergency (A&E)	10	6.0%		
Pharmacy	5	3.0%		
Presented to hospital				
Yes	18	10.7%		
Stayed overnight	7	4.2%		

Cases were significantly more likely to be younger, with those aged under 10 years the most likely to become unwell, compared to those aged 40 years and over (OR: 3.89, 95% CI: 1.98–7.73) (Table 3). No significant difference was observed between males and females, or the duration of time spent in the barn. The odds of developing illness increased with each successive week, with cases significantly more likely to have attended in weeks 2 (OR: 7.41, 95% CI: 3.22–20.19), 3 (OR: 8.79, 95% CI: 3.91–23.57), and 4 (OR: 10.05, 95% CI: 4.43–27.14), compared with week 1. Although photos from the event showed visitors eating and drinking around animals, these exposures had no significant impact on the odds of developing illness at the univariable level.

**Table 3.** Univariable associations between exposures and odds of developing cryptosporidiosis-like illness

	Case		Non-case		Univariable		
	n	%	n	%	OR	95% CI	*p*-value
Age group (years)							
0–9	26	15.6%	27	7.3%	3.89	(1.98–7.73)	<0.001
10–19	12	7.2%	20	5.4%	2.42	(1.04–5.50)	0.036
20–29	23	13.8%	45	12.2%	2.06	(1.07–3.96)	0.029
30–39	56	33.5%	139	37.7%	1.63	(0.98–2.75)	0.065
40+	28	16.8%	113	30.6%	Ref.		
Unknown	22	13.2%	25	6.8%			
Sex							
Male	32	19.0%	72	19.4%	0.97	(0.61–1.54)	0.98
Female	133	79.2%	291	78.2%	Ref.		
Unknown	3	1.8%	9	2.4%			
Time period							
Week 1 (1 March 2024–7 March 2024)	6	3.6%	91	24.5%	Ref.		
Week 2 (8 March 2024–14 March 2024)	43	25.6%	88	23.7%	7.41	(3.22–20.19)	<0.001
Week 3 (15 March 2024–21 March 2024)	62	36.9%	107	28.8%	8.79	(3.91–23.57)	<0.001
Week 4 (22 March 2024–26 March 2024)	57	33.9%	86	23.1%	10.05	(4.43–27.14)	<0.001
Time spent in barn							
Less than 30 min	45	26.9%	101	27.4%	Ref.		
Between 30 min and 1 hour	92	55.1%	204	55.3%	0.98	0.64–1.51	0.981
Over an hour	30	18.0%	64	17.3%	0.99	0.57–1.73	0.93
Lamb contact							
Any contact with lambs	166	99.4%	356	96.7%	5.60	(1.09–102)	0.10
Close contact with lambs^a^	142	88.2%	275	79.5%	1.93	(1.14–3.41)	0.014
Faeces on clothes or skin	50	36.5%	49	14.0%	3.51	(2.22–5.59)	<0.001
Dose response: Level of lamb contact							
Fed without touching or stroked without holding	19	11.8%	71	20.5%	Ref.		
Held the lamb on the lap	38	23.6%	86	24.9%	1.65	(0.88–3.16)	0.121
Cuddled lamb	77	47.8%	146	42.2%	1.97	(1.12–3.58)	0.021
Kissed/nuzzled face with lamb	27	16.8%	43	12.4%	2.35	(1.17–4.77)	0.017
Risk behaviours at the farm							
Ate or drank anywhere on the farm	40	24.4%	114	31.1%	0.71	(0.47–1.08)	0.11
Ate or drank after the barn	57	34.3%	127	35.0%	0.97	(0.66–1.43)	0.88
Hand hygiene							
Washed with water only	15	9.6%	11	3.2%	Ref.		
Used hand sanitizer only	12	7.7%	14	4.0%	0.63	(0.21–1.87)	0.406
Washed with soap and water	129	82.7%	323	92.8%	0.29	(0.13–0.65)	0.003
Hand hygiene continued							
Washed hands after animal contact	133	80.6%	313	86.7%	0.64	(0.39–1.05)	0.072
Washed hands before eating/drinking	133	80.6%	267	74.0%	1.46	(0.94–2.32)	0.10
Paper towels available	102	63.4%	267	76.1%	0.54	(0.36–0.82)	0.003
Preventative messaging							
Aware of handwashing facilities	160	95.8%	354	98.3%	0.39	(0.12–1.18)	0.09
Aware not to eat/drink around animals	18	13.7%	87	36.7%	0.27	(0.15–0.47)	<0.001
Aware of the importance of washing hands after animal contact	40	31.7%	198	68.8%	0.21	(0.13–0.33)	<0.001

Abbreviations: OR, odds ratio; 95% CI, 95% confidence interval.

a Close contact was defined as any one of the following: holding the lamb on your lap, cuddling, or kissing/nuzzling faces with the lambs.

Several exposures within the barn were associated with illness at the univariable level. Close contact with lambs (OR: 1.93, 95% CI: 1.14–3.41) and having faeces on clothes or skin (OR: 3.51, 95% CI: 2.22–5.59) were both associated with an increased odd of developing illness. Thumb-sucking and nail-biting were also associated with an increased odds of becoming unwell; however, after stratifying by age (under 10 years vs. 10 years and over), the effect was reduced and no longer significant. Mobile phone use was negatively associated with becoming unwell; however, when examined in the two age bands, this finding remained a negative association for those aged under 10 years but reversed among those aged over 10 years, but neither was significant.

Washing hands with soap and water was protective against illness (OR: 0.29, 95% CI: 0.13–0.65), compared to using water only. The availability of paper towels (OR: 0.54, 95% CI: 0.36–0.82) was found to be protective, alongside being aware of preventative messaging surrounding the importance of not eating or drinking around animals (OR: 0.27, 95% CI: 0.15–0.47) and washing hands after animal contact (OR: 0.21, 95% CI: 0.13–0.33).

There was also evidence to suggest a dose–response relationship with the level of lamb contact reported. Using the lowest tier of contact as the baseline, we observed that the odds of becoming unwell increased with each successive level of lamb contact. The two highest tiers of contact, cuddling (OR: 1.97, 95% CI: 1.12–3.58) and kissing/nuzzling the lambs (OR: 2.35, 95% CI: 1.17–4.77) significantly increased the odds of developing illness.

Potential confounders for *Cryptosporidium* infection (such as recent foreign travel, having a private water supply, and contact with other animals) were considered, but none were significant.

After controlling for the other variables included in the multivariable model (Table 4), being under 20 years (aOR: 4.50, 95% CI: 1.99–10.30 and aOR: 2.56, 95% CI: 1.00–6.43) was a significant predictor of illness, with the odds of becoming unwell decreasing as age increased. Those with faeces on their clothes or skin (aOR: 3.63, 95% CI: 2.14–6.20) were also more likely to develop illness. Thorough handwashing (with soap and water) was found to be protective (aOR: 0.48, 95% CI: 0.24–0.98), along with eating or drinking anywhere on the farm (aOR: 0.57, 95% CI: 0.32–0.98).

**Table 4.** Multivariable associations between exposures and odds of developing cryptosporidiosis-like illness

	Univariable			Multivariable		
	OR	95% CI	*p*-value	aOR	95% CI	*p*-value
Exposure						
0–9 years	3.89	(1.98–7.73)	<0.001	4.50	(1.99–10.30)	<0.001
10–19 years	2.42	(1.04–5.50)	0.036	2.56	(1.00–6.43)	0.046
20–29 years	2.06	(1.07–3.96)	0.029	1.72	(0.78–3.78)	0.175
30–39 years	1.63	(0.98–2.75)	0.065	1.43	(0.78–2.66)	0.252
Used soap and water	0.29	(0.13–0.65)	0.003	0.48	(0.24–0.98)	0.040
Faeces on clothes or skin	3.51	(2.22–5.59)	<0.001	3.63	(2.14–6.20)	<0.001
Ate or drank at the farm	0.71	(0.47–1.08)	0.11	0.57	(0.32–0.98)	0.048

Abbreviations: aOR, adjusted odds ratio; OR, odds ratio; 95% CI, 95% confidence interval.

## Discussion

This was a large outbreak of cryptosporidiosis. Initially, a total of 67 cases (57 confirmed) were reported during the outbreak investigation. Additional case finding during the analytical investigation identified an additional 135 probable cases (Supplementary Material 2), making it one of the largest reported outbreaks of *Cryptosporidium* in Wales, and the largest to date associated with a farm setting in England and Wales [8]. The epidemiological results, in combination with environmental inspections and microbiological results identifying consistent MLVA profiles among confirmed cases and the lambs used for the event, indicate a continuous source outbreak linked to a common exposure. This supports the primary hypothesis of the outbreak investigation that the lambs used for the event were the source of the outbreak, particularly as the odds of becoming unwell increased with each successive week the event was open, suggesting a build-up of oocysts in the lambs and environment over the period. Almost 40% of identified cases reported seeking healthcare for their symptoms, with 7 (5%) hospitalized at least overnight, and 15% of laboratory-diagnosed cases, demonstrating the substantial healthcare impact associated with this outbreak.

The gp60 subtype (IIaA15G2R1) identified in this outbreak is the most frequently reported *C. parvum* subtype in sheep worldwide [21] and one of the most frequently identified subtypes in *Cryptosporidium* outbreaks in England and Wales, with most of these outbreaks linked to animal contact [8, 13]. The MLVA profiles identified were unique to this outbreak at the time of investigation and have rarely been reported since (CRU unpublished data).

The findings of the analytical study strongly suggested that close contact with lambs was associated with illness. The odds of developing illness increased with each level of contact and were highest among those who cuddled and kissed/nuzzled the lambs, supporting evidence from a previous outbreak linked to a similar event in England [12]. Discussions with the farm owner revealed that lambs often urinated or defecated on visitors when they were held for prolonged periods, and multivariable analysis highlighted that those exposed to faeces on clothes or the skin were significantly more likely to develop illness, suggesting a need to limit lamb contact at these events. Indeed, a recent review of outbreaks linked to animal premises in England and Wales between 2009 and 2019 highlighted that the prolonged exposure associated with bottle-feeding lambs was a risk factor for developing cryptosporidiosis [13]. Similarly, a study examining a large petting-farm outbreak in England where the premises complied with the established Code of Practice noted that lamb contact presented a transmission risk [11], which indicates that more robust infection control measures and standards against which environmental inspections can be assessed will likely help mitigate transmission risks. In this outbreak, animals were housed in the same barn used for feeding events, visitors were encouraged to have a high level of contact with lambs, and sanitation facilities were poor, both of which likely increased the risk of faecal matter contaminating clothing and hands and thus increased transmission risk.

Data from England and Wales indicates *Cryptosporidium* is most common in young children [22]. Those aged under 10 years were the most likely to become unwell in this outbreak, with the odds of developing illness decreasing as age increased. This may, in part, be explained by children often having poorer hand hygiene, being more likely to engage in behaviours that facilitate transmission (such as nail-biting or thumb-sucking) and being less likely to thoroughly wash their hands without appropriate supervision. While thorough handwashing (with soap and water) was found to be protective against illness, sanitation facilities on site were inadequate for their purpose. Inadequate handwashing facilities have been previously highlighted as a contributory factor in cryptosporidiosis outbreaks [10, 23] and, in this outbreak, unclean sanitation facilities without hot water and an adequate supply of soap and paper towels likely contributed to the spread of infection. Event organizers should follow those recommendations pertaining to sanitation facilities outlined in the guidance to reduce the potential for environmental contamination, alongside ensuring children are able to effectively wash their hands after animal contact and are properly supervised during these events.

Evidence from environmental investigations, case reports, and social media photos of the event indicates a lack of awareness and adherence to the Code of Practice at the farm. Several large outbreaks of *Cryptosporidium* associated with working farms diversifying into lamb contact events have been reported in England in 2023–2024, all of which highlighted close contact with lambs and inadequate IPC measures, as well as a high healthcare burden [24]. In May 2025, another large outbreak of *Cryptosporidium* associated with a lamb-feeding event in Wales was reported, resulting in 89 confirmed cases and 16 people requiring overnight hospital care [25]. Current guidance [15] includes clear advice regarding the importance of quarantining sick animals, maintaining cleanliness of animal contact areas, segregating eating areas from animal contact areas, and ensuring the availability of adequate handwashing facilities. In this outbreak, animals were housed in the same barn used for feeding events, eating/drinking within the barn was not prohibited, sanitation facilities were poor, and faecal matter was present on the barn floor during the site visit, indicating improvements are necessary to increase the visibility of the guidance among commercial farms offering public access to livestock, particularly among those who have recently diversified and may not be aware guidance exists. There is, however, little discussion in the Code of Practice surrounding the need to limit close or high-level animal contact at these events besides discouraging visitors, particularly children, from kissing the animals. Although there are clear educational opportunities provided by these events, this study has highlighted the role that close contact with animals at these events can have in increasing transmission risk, suggesting a need to strengthen current guidance to discourage this type of contact.

Although animal-contact events are a well-established risk for *Cryptosporidium* transmission, relatively little is known about the individual behaviour associated with transmission risk. One recent study in England [12] found, similar to the current study, that those who held or cuddled lambs were more likely to develop cryptosporidiosis-like illness, along with those who engaged in habits such as nail-biting and thumb-sucking. They also noted inadequate sanitation facilities and environmental contamination resulting from housing animals in the barn used for petting. In another outbreak in Scotland [23], authors noted direct contact with scouring lambs and poor handwashing facilities as contributory risk factors for the outbreak. Our study did not find evidence of the importance of handwashing before eating previously reported [11], but we did find a similar protective effect associated with promoting good hand hygiene and risk awareness [11, 12].

The initiative of the farm to close the event and request a veterinary visit, and the fact that a faecal sample from the lambs was retrieved quickly – facilitated by APHA to be sent for analysis at the CRU – are commendable and provided strong evidence to identify the transmission source in this outbreak, highlighting the value of multi-agency collaboration during outbreaks. The submission of all positive human and animal samples for species identification and subtyping should be encouraged, given the valuable evidence and insight this provides. The farm engaged well with public health teams and has since significantly improved its procedures in preparation for upcoming lamb-feeding events, aligning itself with current guidance and limiting close contact with lambs following study recommendations.

## Limitations

Several limitations should be considered when interpreting the findings of this study. First, the nature of the booking system meant that an accurate figure for the total number of attendees to the farm over the study period was not available. As contact details for all visitors were not available, we used the online booking system to recruit the cohort. The limitation of this approach is that each booking was completed by a single individual, but may represent the attendance of several visitors. Although we asked the booking party to distribute the questionnaire to everyone in their booking, we cannot be assured that everyone who attended received or completed a questionnaire. As a result, we used ORs in place of risk ratios as the measure of association. Second, the cohort study employed a sensitive case definition, and without microbiological confirmation, there is no way to know whether those cases included represent a true *Cryptosporidium* infection or an alternative gastrointestinal illness (although those included as cases are consistent with those who would have been classified as ‘probable’ cases during the original outbreak investigation, with symptoms consistent with cryptosporidiosis). Third, repeated exposure to *Cryptosporidium* (due to, e.g., age, occupation, or residence) has been shown to result in less severe symptoms of a shorter duration, with repeat episodes declining as age increases [26]. In this outbreak, infection among adults may have been attenuated by prior exposure, resulting in some misclassification of cases with mild symptoms. However, assessing this effect was beyond the scope of this study. Finally, all questionnaires are subject to recall bias. Responses received ranged between 4 and 8 weeks post-exposure, which may have impacted the accuracy of responses, a limitation further affected through the reliance of parents/guardians to accurately complete the questionnaire on behalf of children. Similarly, social desirability bias may have led to inflated accounts of protective behaviours such as thorough handwashing.

A review of the outbreak case data during this study led to the exclusion of seven symptomatic individuals (two of whom were confirmed to have a MLVA profile consistent with outbreak cases) as they did not fit the case definition set during the outbreak investigation by the IMT. Six of these cases reported an incubation period of 2 days, suggesting the incubation period set for the case definition (3–14 days) may have been overly restrictive, and case counts may have been higher.

## Conclusion

This investigation highlights the risk posed by close contact with lambs at feeding events, particularly for children. It involved a high healthcare burden and underscores the potential for large *Cryptosporidium* outbreaks at lamb-feeding events where appropriate IPC measures are not adopted. There is a need to improve awareness of, and adherence to, established codes of practice among those commercial farms diversifying into public events with livestock; however, our findings also indicate a need to strengthen this guidance with recommendations to limit close animal contact at lamb-feeding events. Development of cryptosporidiosis-like illness following animal contact in farm settings is well documented, and the growing popularity of these events comes with an increased risk of similar outbreaks. Improvements should be considered to ensure these events reflect the learning from previous outbreaks.

### Public health recommendations

Given the association between close contact with lambs and the increasing likelihood of developing illness, visitors, particularly children, should be discouraged from very close contact, such as holding, cuddling, kissing, or nuzzling lambs, at feeding events. Current guidance should be strengthened to limit this type of contact, and event organizers should consider keeping lambs enclosed in a pen, with visitors encouraged to feed them via a bottle from the other side of the enclosure.

Awareness of and adherence to current guidance among commercial farms offering public access to livestock needs to be improved, alongside improving general risk awareness among both event staff and visitors of the risks posed at animal contact events. Future research may benefit from qualitative studies to investigate the perception of risk posed by animal contact events among both visitors and staff to understand why awareness of current guidance has declined.

Faecal contamination was a significant predictor of illness in this outbreak, likely exacerbated by visitors’ close contact with lambs. Event organizers should ensure that scouring lambs are quarantined, any areas designated as animal contact areas are disinfected regularly, and decontamination of clothing is encouraged. Handwashing facilities should include hot and cold running water, soap, and paper towels, and their use should be encouraged verbally and through signage. Children, in particular, should be supervised to ensure thorough handwashing. The use of disposable clothing covers may be considered to limit the spread.

Although the emergence of online booking systems and social media to advertise animal contact events has likely increased their accessibility and visibility, they also present the opportunity to promote public health advice and improve risk awareness. Event organizers may consider incorporating public health advice within booking confirmations and on their social media profiles during events.

## Supporting information

10.1017/S0950268825100198.sm001 Jones et al. supplementary material Jones et al. supplementary material

## Data Availability

Data are available on reasonable request to the authors. Restrictions may apply to the availability of personal data linked to patient and study participant information.

## Supplementary material

The supplementary material for this article can be found at http://doi.org/10.1017/S0950268825100198.
