# A large *Cryptosporidium parvum* outbreak associated with a lamb-feeding event at a commercial farm in South Wales, March-April 2024: a retrospective cohort study – ERRATUM

**DOI:** 10.1017/S0950268825100435

**Published:** 2025-08-29

**Authors:** Gethin Jones, Joshua Matizanadzo, Andrew Nelson, Rachel M. Chalmers, Daniel Rhys Thomas, Stuart Williams, Maria Pinch, Alison Sykes, Rhianwen Stiff, Chris Williams

**Affiliations:** 1 UK Field Epidemiology Training Programme, UK Health Security Agency, London, UK; 2 Communicable Disease Surveillance Centre, Public Health Wales, Cardiff, UK; 3 Cryptosporidium Reference Unit, Public Health Wales, Swansea, UK; 4 Food Safety, Health and Safety and Communicable Disease Control Team, Caerphilly County Borough Council, Caerphilly, UK; 5 Health Protection Team, Public Health Wales, Cardiff, UK

When this article was published in Epidemiology and Infection it contained an error in the Author Contributions section. This was due to the incorrect implementation of the author’s corrections during article proofing. The Author Contributions should be as follows:
