# Supplementary material for: A large Cryptosporidium parvum outbreak associated with a lamb-feeding event at a commercial farm in South Wales, March–April 2024: a retrospective cohort study
Source: Epidemiol Infect. 2025 Jul 14;153:e82. doi: 10.1017/S0950268825100198 (PMC12308611; doi:10.1017/S0950268825100198)
Supplement: Jones et al. supplementary material [file S0950268825100198sup001.docx]

| **Outbreak investigation** |
| --- |
| Public Health Wales along with partner agencies is investigating an outbreak of illness amongst people who visited Farm X between 1st March 2024 and 26th March 2024.  In order to understand the source of the outbreak, we would like anyone who attended the farm to complete a questionnaire.  We have identified that you booked a lamb feeding session at this farm or someone booked on your behalf. We need a response to the questionnaire for each person who visited the farm, whether or not they were unwell.  All complete questionnaires and associated data will be held in line with current Public Health Wales guidance. Further information on how Public Health Wales processes your data can be found here: Privacy Notice |
| **Farm attendance** |
| Did you attend Farm X on or between 1st March 2024 and 26th March 2024?   - Yes - No |
| **Questionnaire receipt** |
| How did you receive this questionnaire?   - Directly from Public Health Wales - From someone else |
| If you did not receive this questionnaire directly from Public Health Wales, who sent it to you?   - Please provide their name - Please provide their email address: |
| **Demographic** |
| Are you answering for yourself or someone else?   - Yourself - Someone else |
| **Yourself** |
| - Please provide your name - Date of Birth (DOB)   - (Date/Month/Year for example 09/02/1976) - What is your sex? - Email address - Your contact number (including area code) - Please list your postcode - What is your ethnicity? - It may be helpful for us as part of this investigation to get back in touch with you, are you happy for us to contact you?   - Yes   - No |
| **Completing on behalf of someone else: Your details** |
| - If you are completing this questionnaire on behalf of someone else please provide your name - Provide your email address |
| **Completing on behalf of someone else: Their details** |
| - Please provide their name - Date of Birth (DOB) - What is their sex? - Email address - Their contact number (including area code) - Please list their postcode - What is their ethnicity? |
| **Completing on behalf of someone else** |
| - It may be helpful for us as part of this investigation to get back in touch with you, are you happy for us to contact you?   - Yes   - No |
| **Please answer the rest of the questionnaire describing your experience, or if you are completing this questionnaire on behalf of someone else, their experiences.** |
| **Symptoms** |
| - Did you experience any of the following symptoms in the 14 days following your visit to the farm? (diarrhoea/runny poos, vomiting/sickness, nausea/feeling sick, abdominal pain/tummy ache)   - Yes   - No |
| - What symptoms did you experience?   - Diarrhoea/runny poos   - Vomiting/sickness   - Nausea/feeling sick   - Abdominal pain / tummy ache   - Fever   - Loss of appetite   - Other, please specify: - If you experienced diarrhoea/runny poos – was it watery?   - Yes   - No - Approximately when did your symptoms start? - Are you still experiencing symptoms?   - Yes   - No - If no, when did your symptoms stop? - Did you seek medical help or care as a result of your symptoms?   - Yes   - No |
| - Which of the following did you use?   - Pharmacy NHS direct (111)   - GP   - A&E   - Admitted to hospital - If you were admitted to hospital, how many nights did you stay?   - I did not stay overnight   - 1 night   - 2 nights   - 3 nights   - More than 3 nights - Have you provided a stool /poo sample for testing?   - Yes   - No - If you are still symptomatic/ill (or have been in the last 14 days) and have not been tested, Environmental-Health colleagues may contact you to arrange testing (you will be given a pot and asked to provide a stool/poo sample). If this applies to you, please provide your name and contact details |
| **Activities** |
| - What was the date of your visit to the farm? (if you visited multiple times, please indicate your most recent visit) - What time did your visit start? (24 hour) - Did you go into the barn where the lambs were kept?   - Yes   - No - How long did you spend in the barn?   - Less than 15 minutes   - Between 15 and 30 minutes   - More than 30 minutes but less than 1 hour   - An hour or over |
| While in the barn did you do any of the following:   - Did you eat or drink anything? - Did you chew gum or similar? - Did you suck your thumb / or bite your nails? - Did you use your phone? (e.g. taking pictures calls browsing just on the table etc.) - If a young child, did they use a dummy? - Did you milk the artificial cow?   - Yes   - No   - Unsure |
| - Did you have any contact with the lambs?   - Yes   - No - How did you feed the lambs?   - Bottle   - By hand (pellets) - How many lambs did you feed?   - 1   - 2   - 3   - More than 3 - What kind of contact did you have with the lambs (please indicate as many as necessary)   - Only held a feeding bottle out to the lamb but did not touch them   - Touched the lamb without holding the lamb or having lamb on your lap   - Held the lamb or had lamb on your lap   - Stroking / petting   - Cwtching/cuddling   - Kissing   - Nuzzling face   - Other, please specify: - Did you enter the lamb pens?   - Yes   - No   - Unsure |
| **Dry food and other animal contact** |
| - Did you feed any animals with the dry food (pellets)?   - Yes   - No - If yes, which animals did you feed?   - Goats   - Calves - Did any animal defecate (poo) on you?   - Yes   - No   - Unsure - Did you notice any animal faeces (poo) on your clothes or skin?   - Yes   - No   - Unsure - Did you eat or drink anything at the farm after leaving the barn?   - Yes   - No - When you were in the barn, was there a member of staff (i.e., someone from the farm) supervising the lamb feeding/cuddling activities?   - Yes   - No |
| **Handwashing** |
| - Did you know where the handwashing facilities were?   - Yes   - No   - Unsure - How did you find out about the hand washing facilities?   - I was told   - I saw signage   - I saw handwashing facilities - Were any of the following available? - Was there soap available? - Was the water hot? - Were there paper towels available to dry your hands? - Did you use the handwashing facilities provided after any of the following?   - After contact with animals   - When leaving the barn   - Before eating or drinking - How did you clean your hands?   - Hand sanitizer   - Water only   - Soap and water - In your opinion, were the handwashing facilities clean?   - Not at all clean   - Slightly clean   - Moderately clean   - Very clean   - Extremely clean |
| **During your farm visit** |
| - During your farm visit, did you do any of the following?   - Did you purchase milkshake/milk products on the premises?   - Did you purchase any food from the pizza place/farm shop?   - Did you use the toilet?   - Did you eat or drink anything while at the farm? - During your farm visit, were you made aware (either by being told or through signage) about the importance of avoiding eating/drinking while interacting with animals? - During your farm visit, were you made aware (either by being told or through signage) about the importance of washing your hands after interacting with the animals? - When you removed your footwear/clothes (worn in the barn) did you wash your hands (with hot water and soap) afterwards? |
| **Other contact outside the farm** |
| Did you experience any of the following symptoms in the 14 days following your visit to the farm? (diarrhoea/runny poos, vomiting/sickness, nausea/feeling sick, abdominal pain/tummy ache) |
| Did you have direct contact with any of the animals listed below? (outside of the farm)   - Pets - Other farm animals - Wild Animals |
| **Symptomatic** |
| In the 14 days before you become unwell, did you do any of the following?   - Did you visit any other farms? - Did you go swimming in a swimming pool river lake or reservoir? - Did you travel overseas? - Drink water from a private water supply? - Eat/drink uncooked leafy vegetables? (e.g. spinach herbs lettuce) - Eat/drink uncooked berry fruits? - Eat/drink unpasteurised milk products or dairy products?   - Yes   - No   - Unsure |
| Did anyone else in your household (besides the person you are completing this form on behalf of) have any of the following symptoms in the 14 days before you became unwell? (Diarrhoea, vomiting, abdominal cramps, nausea)   - Yes - No |
| **Non-symptomatic** |
| In the week before your visit to the farm, or the week after your visit to the farm, did you do any of the following? |
| **Anything else** |
| Do you have anything else to share that you think may be relevant? |
| Thank you very much for your time.  If you have any questions, please email: |
